# The Molecular Biology of Vestibular Schwannomas and Its Association with Hearing Loss: A Review

**DOI:** 10.1155/2012/856157

**Published:** 2012-02-20

**Authors:** Erika Celis-Aguilar, Luis Lassaletta, Miguel Torres-Martín, F. Yuri Rodrigues, Manuel Nistal, Javier S. Castresana, Javier Gavilan, Juan A. Rey

**Affiliations:** ^1^Instituto Nacional de Neurología y Neurocirugía Manuel Velasco Suarez, 14269 Ciudad de México, DF, Mexico; ^2^Department of Otolaryngology, “La Paz” University Hospital, 28046 Madrid, Spain; ^3^Unidad de Investigación, Laboratorio Oncogenetica Molecular, “La Paz” University Hospital, 28046 Madrid, Spain; ^4^Department of Pathology, “La Paz” University Hospital, 28046 Madrid, Spain; ^5^Brain Tumor Biology Unit, CIFA, University of Navarra School of Sciences, 31009 Pamplona, Spain

## Abstract

Hearing loss is the most common symptom in patients with vestibular schwannoma (VS). In the past, compressive mechanisms caused by the tumoral mass and its growth have been regarded as the most likely causes of the hearing loss associated with VS. Interestingly, new evidence proposes molecular mechanisms as an explanation for such hearing loss. Among the molecular mechanisms proposed are methylation of TP73, negative expression of cyclin D1, expression of B7-H1, increased expression of the platelet-derived growth factor A, underexpression of PEX5L, RAD54B, and PSMAL, and overexpression of CEA. Many molecular mechanisms are involved in vestibular schwannoma development; we review some of these mechanisms with special emphasis on hearing loss associated with vestibular schwannoma.

## 1. Introduction

Vestibular schwannomas (VSs) can be classified into two broad groups: unilateral sporadic vestibular schwannoma and those associated with neurofibromatosis type 2 (NF2). VSs constitute 8% of all benign intracranial tumors, and sporadic unilateral schwannomas represent up to 95% of all VSs [1]. As new population-based studies are performed, the true incidence of VS appears to be higher than expected [2–5]. A nationwide study performed in Denmark [2] revealed that the incidence of VS had been rising from 5 cases per million population per year in 1977–1981 to 10 cases in 1992–1995. In 2004, the same research group estimated an incidence of 11.5 cases per million inhabitants per year during a 25-year period (1976–2001) [3]. Data from a US national tumor registry (2010) reported a VS incidence rate of 1.1 cases per 100,000 people per year [4]. On the other hand, Evans et al. found an incidence of 1 case in 80,000 individuals for sporadic VS, and 1 in 70,000 if NF2-related tumors were included [5]. These increasing numbers are probably due to the effect of newer and more sensitive diagnostic tests, especially magnetic resonance imaging (MRI). The age of presentation of VS is usually the fourth and fifth decades. Even though a benign tumor, if large enough, can cause neurological symptoms like hydrocephalus, brainstem compression, herniation, and ultimately death. 

NF2 is an autosomal dominant disease representing 5% of all VSs. Patients with NF2 are characterized by having bilateral vestibular schwannomas. Half of these patients do not have a family history of the disease [1] and therefore represent new germline mutations. The Manchester criteria for the diagnosis of NF2 have been described elsewhere [6, 7]. These patients can also present other intracranial benign tumors. There are three types of NF2, distinguished according to clinical presentation and severity: Wishart type, Gardner type, and mosaic NF2. The Wishart type appears in childhood or late adolescence and consists of bilateral vestibular schwannomas associated with spinal tumors. The Gardner type appears later in life and is less debilitating, with patients developing bilateral vestibular schwannomas but few meningiomas. Mosaic NF2 occurs when a postzygotic mutation takes place and only a portion of the cells carry this mutation. Around 25% of NF2 patients with apparently healthy parents have a mosaicism [8].

Schwannomatosis has been recently defined as a new form of neurofibromatosis. It consists of multiple schwannomas without associated vestibular schwannomas [1]. The main symptom of affected patients is pain. The SMARCB1 gene has been found to be mutated in schwannomatosis patients [9–12]. SMARCB1, located in chromosome band 22q11.2 [9–12], is a tumor suppressor gene that encodes the INI1 protein. Germline mutations have been described in both familial [10–12] and sporadic [9, 11, 12] cases of schwannomatosis. Immunohistochemical analysis of VS samples [12] detected a mosaic pattern of INI1 expression in 93% of familial schwannomatosis patients, 55% of sporadic schwannomatosis cases, 83% of NF2-associated tumors, and 5% of solitary sporadic schwannomas. These findings suggest that the SMARCB1 gene might also play a role in NF2 tumors [12].

It is estimated that 80% of patients with VS initially complain of hearing loss or tinnitus. Such hearing loss could be the result of various mechanisms [13]. Among them are direct compression of the cochlear nerve by the tumor; occlusion or vascular compression of the internal auditory artery; intratumoral bleeding; biochemical changes in the inner ear, caused by vascular compression or internal auditory canal occlusion. Another cause that has emerged as an interesting possible explanation of this symptom relates to molecular data. Some authors [13, 14] suggest that a degeneration of the inner ear may be caused by a toxic substance produced by the tumor, or by a deficiency in a factor that is crucial for proper inner ear function. Dissociation between tumor size and hearing loss has been described elsewhere [13, 15] and could be an objection to the theory that compression is the sole cause of hearing loss. New ways to explain deterioration of hearing in patients with VS are needed.

The molecular biology of VS has been explained by several pathogenic mechanisms including NF2 gene mutation [1, 16, 17], chromosome 22 loss [16–20], NF2 gene mitotic recombination [18, 20], DNA methylation [21, 22], deregulation of genes [23], immunogenic factors [24, 25], cytokines, and growth factors [26–30]. Early data on the genetic alterations of VS were provided by cytogenetic studies [31–37]. Monosomy 22 was identified in various types of schwannomas, including VS. The incidence of total loss of chromosome 22 varies among VS studies and can reach 50% [31–37]. Other chromosome changes have been observed rarely, and, although these variations do not show a consistent pattern, losses of chromosomes 14, 16, 17, 18, and Y have been observed in at least one sample [31–37]. Warren et al. [38] studied 76 vestibular schwannoma samples, finding that 10% of the tumors showed copy gains in chromosome 9q34. Three tumors had gains in 17q, and, in three or fewer tumors, copy gains and copy losses were identified in chromosomes 10, 11, 13, 16, 19, X, and Y. The relevance of copy gains in chromosome 9 is still under investigation. In parallel, loss of heterozygosity (LOH) studies have demonstrated that deletions of chromosome 22 have occurred in up to 80% of schwannomas, including VS cases. These findings suggest that genes located on this chromosome play an essential role in VS development [39–41]. Allelic losses at 1p have also been described in a few cases [42]. Mitotic recombination consists of deletion followed by reduplication. This mutational mechanism can generate two identical copies of a mutated gene in the absence of a wild-type copy [17]. This mechanism is responsible for LOH in a proportion of schwannoma cases [18, 20] (see Table 1).

## 2. NF2 Gene

The molecular study of VS began in 1993 with the identification of the NF2 gene which contains 17 exons and is located in chromosome 22q12.2 [43, 44]. The coding region of messenger RNA is 1785 base pairs in length and encodes 595 amino acids, producing a protein named merlin (for “moesin-ezrin-radixin-like protein”) or schwannomin (derived from schwannoma). This family of proteins presents an N-terminal globular domain (FERM domain), an *α*-helical stretch, and a charged C terminus at the end [1, 16]. It is believed that this protein acts by linking the actin cytoskeleton to the plasma membrane. Merlin has the ability to change its conformation status. It can fold into itself (closed conformation) or be unfolded (open conformation). This can be achieved by folding its alpha helical portion and c-terminal portion [45]. P21-activated kinase 1 (PAK), a downstream effector of Rac1, promotes the phosphorylation of merlin at S518 with conversion to an open conformation, initiating its degradation [46]. The folded form of merlin is known for its tumor-suppressing properties [47]. The folded version of merlin binds to DCAF1 and suppresses cell proliferation by inhibiting E3 ubiquitin ligase CRL4 [48]. CRL4^DCAF1^ plays a role in DNA replication and, if inactivated by merlin, favors the upregulation of genes related to apoptosis and cell-cycle arrest [48].

Rac1 is a member of the Rho GTPase family and regulates signaling pathways such as MAPK, JNK/SAPK, NF-k*β*, and PI-3K [49, 50]. Rac1 is associated with tumorigenesis [50]. Merlin downregulates Rac1-mediated canonical Wnt signaling, becoming a negative feedback loop preventing Rac1 activation and therefore phosphorylation by PAK [51–53].

There is an association between Merlin, CD44, and *β*1-integrin. CD44 is a transmembrane hyalorunic acid receptor implicated in cell-cell adhesion, cell matrix adhesion, cell motility, and metastasis [1]. Herrlich et al. [54] demonstrated that at high cell density, hypophosphorylation of merlin occurs, which inhibits cell growth. On the other hand, at low cell density, phosphorylation of merlin occurs, becoming growth permissive. In both scenarios, merlin interacts with CD44, promoting cellular contact-dependent inhibition.

The NF2 gene has been shown to be a tumor suppressor gene. This was evident in mouse models, where overexpression of the NF2 gene (merlin) has been proven to limit cell growth in mouse fibroblast and rat schwannoma cells [55, 56].

In various studies, epidermal growth factor (EGF) and epidermal growth factor receptor B (ErbB2) are upregulated in VS cells [57, 58]. Merlin's proliferative activity depends on the regulation of these factors. Neuroglin induces proliferation of VS cells by binding to ERbB2 and ERbB3 [58], as subsequent activation of PI3K and MAPK (mitogen-activated protein kinase) pathways occurs. Both pathways are associated with cellular invasion [27]. MAPK is regulated by mitogen stimuli mixed lineage kinase 3 (MLK3). Finally, merlin has been shown to inhibit MLK3 and epidermal growth factors, demonstrating its tumor suppressive properties [59].

These molecular and signaling pathways can help us to understand new therapies for VS treatment, some of which will be discussed further below.

## 3. Mutations of the NF2 Gene, DNA Methylation, and Hearing Loss

Mutations of the NF2 gene have been found in both NF2 and unilateral sporadic schwannoma patients. More than 200 mutations have been identified to date, including single-base substitutions, insertions, missense, and deletions [60]. NF2 gene inactivation is necessary for VS to grow. According to Knudsen's two-hit hypothesis, in NF2 patients, the germline NF2 allele is inactivated and tumors occur when a wild-type allele is inactivated by allelic loss, silencing, or mutation [17]. On the other hand, sporadic unilateral VS formation is explained by somatic biallelic NF2 inactivation [17]. Regarding the effect of age on unilateral sporadic VS formation, Evans et al. [61] have hypothesized that new somatic mutations are added to the NF2 gene due to impaired DNA repair mechanisms. They also found an increased ratio of somatic frameshift to nonsense mutations with increased age at diagnosis.

Hadfield et al. [20] identified germline mutations in 89% of a sample of 97 patients with an NF2 diagnosis and a second mutational event in 79% of the sample. LOH was the most common form of second hit. Mitotic recombination was the cause of LOH in 14 out of 72 total evaluable NF2 tumours. On the other hand, in a sample of 104 patients with unilateral sporadic VS, 66% had at least one somatic point mutation identified by MLPA (Multiplex ligation dependent probe amplification), loss of heterozygosity (LOH) was found in 56%, and mitotic recombination was a cause of LOH in 6%.

The genotype-phenotype correlation in NF2 patients has been studied. While some authors deny any correlation [1, 62], others affirm it [63, 64]. Recent evidence has detected genotype-phenotype correlation in NF2 patients [65–68]. The Manchester group [65] studied 411 NF2 patients and correlated the presence of meningiomas with gene mutations. Interestingly, they did a genotype-phenotype correlation based not only on the type of mutation but also on the position effect of the mutation itself. Patients with mutations in exons 14 or 15 develop meningiomas less frequently; in other words, patients with mutations located in exons 1 to 13 had a higher risk of developing meningiomas. Regarding the type of mutation, it was observed that individuals with truncating mutations (nonsense or frameshift) had a higher risk of meningiomas than patients with missense or splicing mutations (58% versus 26% versus 35%, resp.). This finding had already been described by Selvanathan et al. [66] and Evans et al. [67], where nonsense and frameshift mutations were associated with more severe NF2 symptoms.

Abo-Dalo et al. [68] demonstrated that clinical features in individuals with large intragenic deletions were similar to those in individuals with mutations affecting single or multiple nucleotides. Milder phenotype was seen in deletions affecting 3′exons 15 and 16 of the NF2 gene, corroborating the same finding of Smith et al., where mutation position was fundamental in phenotype expression.

Currently, there is a new understanding of the role played by the type and position of NF2 mutations in the phenotype of the disease. Nevertheless, it has been reported that families and identical twins with NF2 carrying the same mutation can develop different phenotypes [69, 70].

 Besides the type and position of the mutation [71], there are other possible mechanisms that can explain NF2 inactivation [1]. Presence of a modifier gene [72], methylation of the regulatory region [21, 22, 73], posttranscriptional alternative splicing, and differential polyadenylation of the NF2 gene [74] are proposed as possible causes. On the other hand, the NF2 gene promoter area is a region of DNA that facilitates transcription. Welling et al. [75] described how methylation of the regulatory region of the NF2 gene occurs. Both positive and negative cis-acting regulatory elements required for transcription of the NF2 gene have been found on the 5′ flanking region of the promoter. This region is rich in G/C and susceptible to inactivation and methylation.

Robertson [76] covered the DNA methylation process extensively, defining it as a crucial epigenetic modification of the genome that is involved in regulating many cellular processes, including transcription. This epigenetic modification of DNA consists of methylation of cytosine at position C5 in CpG dinucleotides. CpG islands are generally unmethylated. It has been claimed that DNA methylation represses transcription directly, by inhibiting the binding of transcription factors, or indirectly, by recruiting methyl-CpG-binding proteins. Hypermethylation of the promoter-associated CpG islands leads to transcriptional silencing and finally to epigenetic inactivation of tumor suppressor genes [21]. The DNA methylation of VS has not been fully studied. Data on the methylation status of the promoter region of RASSF1A in brain tumors have been reported [77]. Only 10% of schwannoma cases displayed this DNA modification. Kino et al. [73] found methylation of 3 CpG islands in 14 of 23 VS patients. Gonzalez-Gomez et al. [22] and Bello et al. [78] identified the 5 tumor-related genes most frequently methylated in VS cases (THBS1, TP73, MGMT, NF2, and TIMP3). These tumor-related genes were chosen on the basis of their critical cancer-related functions, since they are frequently hypermethylated and silenced in other neoplasms. Among their known functions are tumor suppression, angiogenesis and invasion inhibition, DNA repair, and detoxification. NF2 gene promoter elements showed hypermethylation in 18% of cases, which suggests an alternative mechanism of NF2 gene inactivation. Aberrant methylation of the NF2 gene could be considered a relatively early event, whereas hypermethylation of other tumor-related genes might represent secondary changes [22]. These results were demonstrated to be specifically due to negativity on control tissues (nonneoplastic nerve sheath and brain samples).

To understand the role of TP73 (located at 1p36.3), we have to be aware of its homology, in terms of structure and conformation, with TP53 (located at 17p13.1). Both genes are involved in apoptosis and inhibition of cell growth. It has been proposed that p53 haploinsufficiency has a role in the development of VS [79]. TP73 null mice show specific developmental defects but no spontaneous tumors, giving rise to multiple protein isoforms with opposite biological properties [80]. Ahmad et al. [79] stated that p73 can produce multiple protein isoforms generated by alternative promoters and alternative splicing. Overexpression of p73 in various carcinomas correlates with poor prognosis [80]. Allart et al. [81] suggested that TP73 plays a major role in cellular differentiation and apoptosis in neuronal tissues. Ahmad et al. [79] confirmed this in their study of 34 VS tissues, which found expression of p73 in 41% of the specimens. Furthermore, after transfecting experimental vestibular schwann cells with p73 plasmid and exposing them to ionizing radiation, an increase in early apoptosis, late apoptosis, and necrosis was observed, as compared to a control group.

Lassaletta et al. [21] explored the methylation status of 16 tumor-related genes in 22 unilateral VSs. DNA methylation values of 9 to 27% were found in 12 of the genes tested: RASSF1A, VHL, PTEN, TP16, CASP8, TIMP3, MGMT, DAPK, THBS1, HMLH1, TP73, and GSTP1. The association discovered between TP73 aberrant methylation and hearing loss was remarkable. The corrected hearing thresholds for patients with methylated and unmethylated TP73 genes were 43 dB and 17 dB, respectively (*P* = 0.04). The frequency most affected was 1000 Hz.

Additionally, Lassaletta et al. [21] found that methylation of TP73 had no association with age, clinical growth index, or tumor size. Other clinical findings concerning tumor-related genes included an association of methylation of CASP8 with age and tumor size and an inverse correlation between RASSF1A methylation and clinical growth index.

The scientific relevance of methylation to hearing loss needs further study, as, at present, methylation of TP73 is the only mechanism implied in hearing loss pathogenesis [6].

## 4. Deregulated Genes in Vestibular Schwannoma

Molecular studies are based on the gene expression of tumors. According to new data [1, 19, 82, 83], it is now possible to differentiate one tumor from another in ways unavailable to histopathology. Molecular investigation also suggests that mutations alone cannot explain the diverse behavior of VS. Welling et al. [82] studied 7 vestibular schwannomas by microarray DNA analysis, concluding that 42 genes were significantly upregulated in six of seven tumors studied. Among the upregulated genes were mediators of angiogenesis like endoglin (an endothelial marker of angiogenesis) and osteonectin (a promoter of cell migration). Downregulated genes included an apoptosis-related putative tumor suppressor gene, LUCA-15. The retinoblastoma protein (pRb) is encoded by the RB1 gene. Lasak et al. [84] examined a deregulated signaling pathway, the retinoblastoma protein (pRb)-cyclin-dependent kinase (CDK) pathway, which was downregulated in 7 of 8 tumors. This signaling pathway is involved in the G1 to S cell cycle progression, promoting cell proliferation. As already stated by Cayé-Thomasen et al. [23], the available data for the CDK pathway is conflicting, since one group found downregulation [84] while the other found upregulation [23]. Gonzalez-Gomez et al. [22] had previously suggested an inverse link between the methylation of RB1 and p16^INK4a^ (found in 15% of samples) and cell cycle regulation, with the latter being altered through epigenetic changes. This in turn may explain the RB1-CDK pathway deregulation [84].

**Figure 1:**
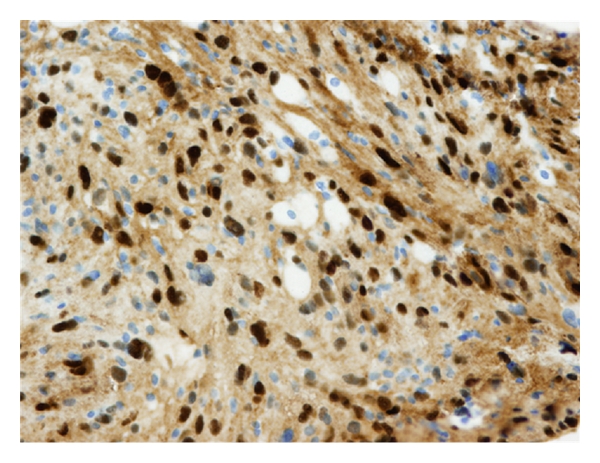
Positive immunostaining (+++) for cyclin D1.

Deregulated expression of growth regulatory genes might play a role in VS progression. Cyclin D1 is a cell-cycle regulatory protein for the mammalian G1-S phase transition and is implicated in cell proliferation and differentiation. Lassaletta et al. [85] found, using immunohistochemistry, cyclin D1 expression in 52% of their cases. This deregulated gene was more frequently present in tumors with nuclear degenerative changes (Figure 1). Patients with negative cyclin D1 expression had a longer duration of deafness (*P* = 0.02) and higher 2,000 Hz hearing thresholds (*P* = 0.04) than cyclin positive patients. In spite of the need for more research to fully understand these results, this is the only study that has made a correlation between clinical symptoms and cyclin deregulation.

By contrast, in a 2006 study, Neff et al. [86] found no staining of cyclin D1 in 15 VS specimens. Stankovic et al. [14] collected VS surgical specimens from 13 patients and classified them into two groups, one with good hearing (word recognition >70% and pure tone average ≤30 dB) and another with poor hearing. The entire genome expression was tested by microarray technology. The expression of selected genes was validated using real-time quantitative reverse transcription-polymerase chain reaction and immunohistochemistry. A chromosomal region (3q27) was found to be expressed differently in the two groups of patients. The peroxisomal biogenic factor 5-like gene (PEX5L), a gene within this chromosomal region with a recognized role in hearing, was found to have underexpression in VS patients with poor hearing. Another 3 genes were found to be underexpressed in VS patients with poor hearing: RAD54, homolog B (RAD54B), and the prostate-specific membrane antigen-like gene. In contrast, carcinoembryonic antigen was highly expressed. The negative results were also of interest: no correlation was found between the presence of axons and preservation of hearing, nor between vessel density and hearing loss. Finally, no significant differences in platelet-derived factor 4 expression were found between the groups.

Stankovic et al. [14] also explained each of their positive results and their possible roles in hearing loss. PEX5L, located in the chromosomal region 3q27, is generally expressed in brain tissue, and it is linked to the regulation of peroxisomal protein import. It is believed that peroxisomal dysfunction could aggravate hearing loss in patients with VS, due to underexpression of PEX5L. The authors suggest pathologic accumulation of fat and/or demyelination and neurodegeneration of the acoustic nerve as possible mechanisms of such hearing loss. They argue that hereditary demyelinating disease or peroxisomal disorders manifest with sensorineural hearing loss similar to that of VS. Moreover, histopathology has also shown demyelinating changes in the nearby vestibular nerve. All of these factors offer support for the theory of peroxisomal dysfunction.

RAD54B is another gene that is underexpressed in patients with VS and poor hearing [14]. This gene has been associated with the recombinational repair of DNA damage. Stankovic et al. found a nuclear distribution of RAD54B in VS patients with poor hearing and cytoplasmic distribution of this same gene in the good hearing group. The authors suggest this may be due to different ways of responding to DNA damage. Nonetheless, a question still remains: how does an impaired ability to repair DNA in VS patients contribute to hearing loss? Mutations in RAD54B have also been identified in non-Hodgkin lymphoma and colon cancer [87], and their relationship to VS development must be fully determined.

PSMAL (glutamate carboxypeptidase III) also showed lower levels of expression in the group with VS and poor hearing [14]. This gene has been reported to have a high expression in brain tissue. Unfortunately, its biological implications for VS remain unknown. On the other hand, the CEA-CAM7 gene and CEA protein had high expressions in VS patients with poor hearing. This differs from the overall results, in which underexpression was the rule. Likewise, high levels of CEA in the cerebrospinal fluid (CSF) have been associated with benign and malignant tumors of the central nervous system (CNS), and it has been proposed that they are also associated with hearing loss [88]. Recently, a new mammalian protein, a member of the carcinoembryonic antigen-related cell adhesion molecule (CEACAM), has been identified [89]. CEACAM16mRNA is expressed in outer hair cells, and its product localizes to the tips of the tallest stereocilia and the tectorial membrane. According to Zheng et al. [89], this localization might imply a role in maintaining the integrity of the tectorial membrane as well as the connection between the outer hair cell stereocilia and the tectorial membrane, which is essential for mechanical sound amplification. Furthermore, a mutation in CEACAM16 leads to autosomal dominant nonsyndromic deafness. These data could clarify the relationship between CEA and hearing loss. Again, more studies in this area are needed.

Stankovic et al. [14] also found a possible link between CEA levels and peroxisomal dysfunction. The authors explained that the activation of the nuclear hormone peroxisome proliferator-activated receptor gamma induces CEA. RAD54B and PSMAL could play an indirect role in the degeneration of the inner ear. According to these authors, this role might be a decrease in the production or responsiveness required for normal auditory nerve and inner ear function.

Using microarray gene expression technology, Lassaletta et al. [90] studied tumor samples surgically removed from 11 patients with unilateral vestibular schwannomas. The expression of platelet-derived growth factor A (PDGFA) was inversely correlated with hearing loss (*r*_*s*_ = − 0.942,   *P* < 0.001). Mean PDGFA expression for tumor patients with <40 dB and ≥40 dB pure tone threshold was 0.73 and 0.56, respectively. PDGFs are mitogenic factors for smooth muscle cells and also act as paracrine growth factors that mediate epithelial-mesenchymal interactions in various tissues. Increasing evidence suggests they may have a role in signaling pathways with a tumor angiogenesis effect. There are 4 members of PDGF (A, B, C, and D), and these bind to two tyrosine kinase receptors that are specific to each member. PDGF A, B, and C bind to PDGFR*α*, and PDGFB and D bind to PDGFR*β*. Ultimately, activation of these pathways leads to cellular responses such as proliferation and migration. The authors had no explanation for the relation between hearing loss and PDGF.

## 5. Immune Response, Vestibular Schwannomas, and Hearing Loss

The literature contains studies of the immunogenic potential of tumors and recently that of the vestibular schwannomas. Rossi et al. [91] described the presence of macrophages, CD8 and CD4 lymphocytes, in VS. Leukocyte migration inhibition has been measured in serum, CSF, and perilymph in VS patients [92]. Archibald et al. [24] proposed that B7 homolog 1 (B7-H1) was a protein aberrantly expressed in malignant tumors (renal, breast, lung, and head and neck cancer) and also in VS. It is reported that B7-H1 acts as a ligand that interacts with its counterreceptor, programmed death-1 (PD-1) on activated T cells, inducing apoptosis and inhibiting their proliferation and cytokine production. As a consequence, there is a diminished immune response to tumor cells and unrestricted growth. Archibald et al. [24] studied 48 VS samples and correlated them with clinical data and with immunohistochemical staining of B7-H1. Patients with failure of tumor control after stereotactic radiation therapy were significantly more positive (*P* = 0.029) in B7-H1 staining. This finding suggests a role for B7-H1 in immune evasion and might explain the continued growth of VS despite radiotherapy. Additionally, patients with worse hearing at the moment of surgery tended to stain more strongly for B7-H1 than the better hearing patients, although no significant difference between both groups was found.

## 6. Cytokines and Growth Factors

Identification of growth factors implied in VS progression can provide new treatment options. Different authors have studied Ki67, proliferation cell nuclear antigens, nerve growth factor receptors, transforming growth factors, fibroblast growth factors, interleukin 6, and hormones [25–30]. Nonetheless, these tumor growth factors have not been identified as independent causes of hearing loss.

Vascular endothelial growth factors (VEGFs), on the other hand, are associated with hearing loss and are highly expressed in vestibular schwannoma [93–95]. Cayé-Thomasen et al. [94] demonstrated a correlation between the concentration of vascular endothelial growth factors in VS samples and rate of tumor growth. Plotkin et al. [95] found VEGF expressed in 100% of 21 NF2-related schwannomas and 22 sporadic schwannoma samples. Moreover, they found hearing improvement in 4 of 7 patients under treatment with Bevacizumab. Bevacizumab is a VEGF neutralizing antibody approved by the FDA for the treatment of cancers. This finding supports the idea that VEGF plays a role in tumor growth and also suggests a possible treatment for hearing loss in VS patients [96].

## 7. Novel Therapies in VS Treatment

Bevacizumab is the most studied agent of the new therapies for vestibular schwannoma [16]. It consists of a humanized monoclonal IgG1 antibody against VEGF. As previously stated, vascular endothelial growth factors are associated with tumor growth and therefore represent a new target in VS therapy.

 Recently, there has been increased attention to the auditory benefit registered in patients treated with Bevacizumab [95, 97]. Mautner et al. [97] treated 2 patients, one for 3 months and one for 6 months. Improved hearing could be registered in the patient treated for 6 months. The only side effect mentioned was high blood pressure in one of the patients.

Plotkin et al. [95] reported the best auditory benefit described in the literature. They administered Bevacizumab to 7 patients, of whom 4 (57%) reported hearing improvement. The results were evaluated on the basis of the word recognition score (WRS). Patient number 2 had the best result, with a WRS of 8% prior to therapy and 98% after therapy. Hearing improvement was sustained for up to 16 months. The authors explained the improvement in hearing as due to reduction of intraneural edema and reduction in tumoral size. This conclusion was based on the evolution of hearing loss, the correlation between the mean apparent diffusion coefficient (a measurement of the magnitude of diffusion of water molecules within tissue and a marker of edema on imaging MRI), tumor shrinkage, and measurements (on a dynamic contrast MRI) of the changes in intratumoral vascular permeability.

Both of these previous studies corroborated a reduction in tumoral burden. In another study published in 2010 [98], the authors demonstrated that anti-VEGF therapy normalizes vasculature in schwannoma xenografts in nude mice and controls tumor growth. Vascular normalization in benign tumors is an important issue when considering this treatment. New anti-VEGF agents are being developed (e.g., PTC299), which have mitigation of hearing loss as a main clinical outcome [16, 99].

Erlotinib is an oral EGFR tyrosine kinase inhibitor. Its main target is the arresting of the proliferative properties of the tumor. Plotkin et al. [100] evaluated 10 patients who underwent treatment with Erlotinib. In three patients, the disease was stable, as measured by radiographic evaluation. Regarding their hearing outcomes, one patient presented a transient hearing response, 2 experienced minor hearing response (lasting 19 and 24 months), 3 patients were stabilized, and 2 presented progressive hearing loss. The authors commented that Erlotinib may have more cytostatic properties as well as being less effective in progressive VS. More studies are needed with emphasis on time to progression as an important outcome, rather than tumoral volume or hearing status.

An in vitro study by Ammoun et al. [101] included VS samples and identified overexpression and activation of EGFR family receptors. They found that lapatinib inhibited ErbB2 phosphorylation and downstream ERK1/2 and AKT activation, resulting in decreased proliferation. Phase II studies are pending.

Other pathways such as PAK inhibitors are under investigation [99].

## 8. Conclusions

In this paper we describe some of the molecular mechanisms involved in vestibular schwannoma development. The NF2 gene mutation, chromosome 22 loss, NF2 gene mitotic recombination, DNA methylation, deregulation of genes, immunogenic factors, cytokines, and growth factors are the key to understand the molecular pathophysiology of VS.

**Figure 2:**
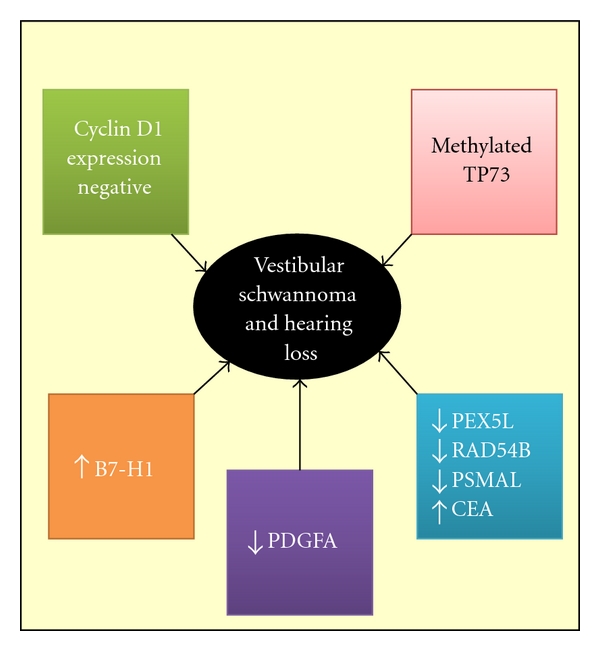
Available evidence of molecular mechanisms implicated in VS patients with hearing loss PEX5L (peroxisomal biogenesis factor 5-like gene), RAD54B (RAD54 homolog B), PSMAL (prostate-specific membrane antigen-like), CEA (carcinoembryonic antigen), PDGFA (platelet-derived growth factor A, B7-H1 (B7 homolog 1).

Also, many theories have been advanced to explain hearing loss associated with VS patients, but there is growing evidence concerning molecular-based data. The methylation of TP73, the negative expression of cyclin D1, the positive expression of B7-H1, increased expression of platelet-derived growth factor A, underexpression of PEX5L, RAD54B, and PSMAL, and overexpression of CEA are factors associated with hearing loss and VS (Figure 2).

Novel therapies also confirm that molecular investigation could be a promising alternative for the treatment of VS. More studies are needed to corroborate these results and, more broadly, to establish links between molecular and clinical data.

## Figures and Tables

**Table 1:** Molecular mechanisms described in VS growth.

NF2 gene mutation
Loss of chromosome 22
NF2 gene mitotic recombination
DNA methylation
Deregulation of genes
Immune response alteration
Growth factors and cytokines
